# Retraction Note: Stress-induced epinephrine promotes epithelial-to-mesenchymal transition and stemness of CRC through the CEBPB/TRIM2/P53 axis

**DOI:** 10.1186/s12967-026-08128-8

**Published:** 2026-04-27

**Authors:** Zili Zhou, Yan Shu, Haijun Bao, Shengbo Han, Zhengyi Liu, Ning Zhao, Wenzheng Yuan, Chenxing Jian, Xiaogang Shu

**Affiliations:** 1 Department of Gastrointestinal Surgery, Sichuan Provincial People’s Hospital, University of Electronic Science and Technology of China, Chengdu, 610072 China; 2 Department of Gastrointestinal Surgery, Union Hospital, Tongji Medical College, Huazhong University of Science and Technology, Jiefang Road No. 1277, Wuhan, Hubei 430022 China; 3 Department of Endocrinology, Liyuan Hospital, Tongji Medical College, Huazhong University of Science and Technology, Wuhan, 430000 China; 4 Department of Breast Surgery, Henan Provincial People’s Hospital, The People’s Hospital of Zhengzhou University, The People’s Hospital of Henan University, Zhengzhou, 450003 China; 5 Department of Gastrointestinal Surgery II, Renmin Hospital of Wuhan University, Wuhan, 430060 China

**Journal of Translational Medicine (2022) 20:262**

10.1186/s12967-022-03467-8

The Editor-in-Chief has retracted this article at the Authors’ request. After publication, the Authors contacted the journal regarding inadvertent image reuse between Fig. 2E and F this article and Fig. 6K in a previously published article from the same author group [1]. Further checks by the Publisher found that in Fig. S5i, the HCT116 and Lovo P53 lanes for the Control and P53 groups appear highly similar. The Authors have stated that the errors occurred during data processing and the selection of the figures.

All authors agree with this retraction.
